# Relationship of actual laminoplasty opening size and increment of the cross-sectional area based on single-door cervical laminoplasy

**DOI:** 10.1097/MD.0000000000010216

**Published:** 2018-03-23

**Authors:** Xiao-Jiang Yang, Rui-Jun Tian, Xing Su, Shan-bo Hu, Wei Lei, Yang Zhang

**Affiliations:** a Department of Orthopaedic Surgery, Xijing Hospital, The Fourth Military Medical University, Xi’an, Shaanxi; b Chinese PLA General Hospital, Beijing, People's Republic of China.

**Keywords:** cervical spondylotic myelopathy, increment of the cross-sectional area, laminoplasty, opening size, single-door cervical laminoplasty, transverse canal diameter

## Abstract

**Background:**

Amounts of clinic research have been performed to investigate the increment of cross-sectional area in single-door cervical laminoplasty (SDCL). However, no one has taken the effects of surgery drill into consideration.

**Methods:**

A mathematical model was built to investigate the relation of actual laminoplasty opening size (LOS), the transverse canal diameter (TCD), and the increment of cross-sectional area in SDCL). The model was based on geometric analysis on deformation of spinal canal; the relation was derived and characterized as: 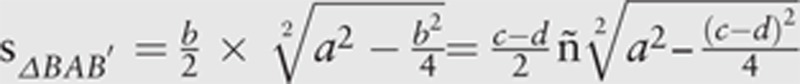, where *a* is the TCD, *b* the actual LOS, *c* the size of mini-plate, and *d* is diameter of the surgery drill bit. In the equation, the related variables would be measured to estimate the increment of cross-sectional area before the surgery. In the current research, 25 patients authorized to use their CT scans of C3∼C7 as the subject samples.

**Results:**

The effects of surgery SDCL were very significant; for each patient, the cross-sectional area was enlarged dramatically after the surgery (*P* < .01). On the contrary, the difference between the cross-sectional area obtained by the equation and that measured by software was statistically negligible (*P* > .05), which confirmed the reliability of the modeling equation.

**Conclusions:**

Before the SDCL, increment of the cross-sectional area can be estimated by the above-mentioned modeling equation with a high-level reliability. This method ensures the optimum selection of mini-plate before operation for each patient.

## 1 Introduction

For decades, single-door cervical laminoplasty (SDCL) has developed to be the primary treatment for syndromes such as ossification of the posterior longitudinal ligament (OPLL), multilevel cervical spondylotic myelopathy (CSM), or multilevel cervical disc herniation associated with developmental spinal canal stenosis.^[1–3]^ Compared to laminectomy, which dominated the surgery treatment for those syndromes before SDCL, SDCL creates more satisfying feedback from patients. Also, SDCL is much more straightforward and convenient for surgery operation.^[4–6]^ Moreover, this technique enables a better recovery for patients, as well as a long-term functionality of the surgery.^[7–10]^

An optimum sagittal canal area is normally the main objective of SDCL. However, the laminoplasty opening size (LOS) plays a key role for the enlargement of sagittal canal. The surgery result would turn out to be undesirable when LOS is either inadequate or excessive.^[11,12]^ Recently, clinical research has been performed to investigate the relation between LOS and the transverse canal diameter (SCD), whereas increasing amount of research have been performed to study the correlation between the LOS and the increment of cross-sectional area.^[1,13,14]^ Meanwhile, among most of the reports on LOS, they neglect the effects of the lamina tissue removed by the drill during the surgery operation, which would result in undesired clinical operation errors. In addition, it is normally non-necessary to consider the effects of transverse canal diameter (TCD) on the increment of the cross-sectional area. It has been verified in the present research that it would substantially enhance the accuracy and reliability of the SDCL when these 2 effects were taken into account at the surgery.

The objective of the present research was to develop a simplified mathematical model for clinical operation of SDCL. The mathematical model precisely characterized the relation of the increment of the cross-sectional area, TCD, and actual LOS. A new concept was introduced for this modeling, which is actual LOS, because details like the drill bit size and TCD were practically taken into consideration at the operation. This method was fully developed and successfully verified by the clinic operations during the present research, which would be applied in the future SDCL for a more efficient and precise operation with high-level reliability.

## 2 Methods

### 2.1 Patient data

The clinical data in the present research were from 25 sample patients (16 men, 9 women) who have undergone SDCL at our institution from January 2016 to February 2017. Among these 25 patients, 7 of them suffered from CSM (5 cervical disc herniation and 2 developmental cervical spinal canal stenosis), while the other 18 of them are diagnosed as OPLL. All 25 patients had received C3-7 laminoplasty. The average age of these sample subjects was 58.3 years (ranging 43–70 years). The median duration history of symptoms was 6.9 months (ranging 5–49 months) before their individual procedure was performed. Note that for each of the sample subjects, conservative treatment had been attempted for 3 months before their SDCL surgery until it had been proved ineffective. Moreover, the magnetic resonance imaging tests confirmed the spinal cord compression caused by cervical disc herniation or spinal canal stenosis at C3-C7 levels.

It is worth noting that in this research all the surgery operations were performed by the same surgeon to ensure the consistence of experimental comparison. The centerpiece mini-plates utilized in this study were from Medtronic (Medtronic Inc., Minneapolis, Minnesota). It was also conditioned that the left side of lamina was set as the lamina opening; thus, the contralateral side plays a role like a hinge to open the lamina. The lamina was cut off with a high-speed surgery hand drill.

### 2.2 Formula derivation

**Figure 1:**
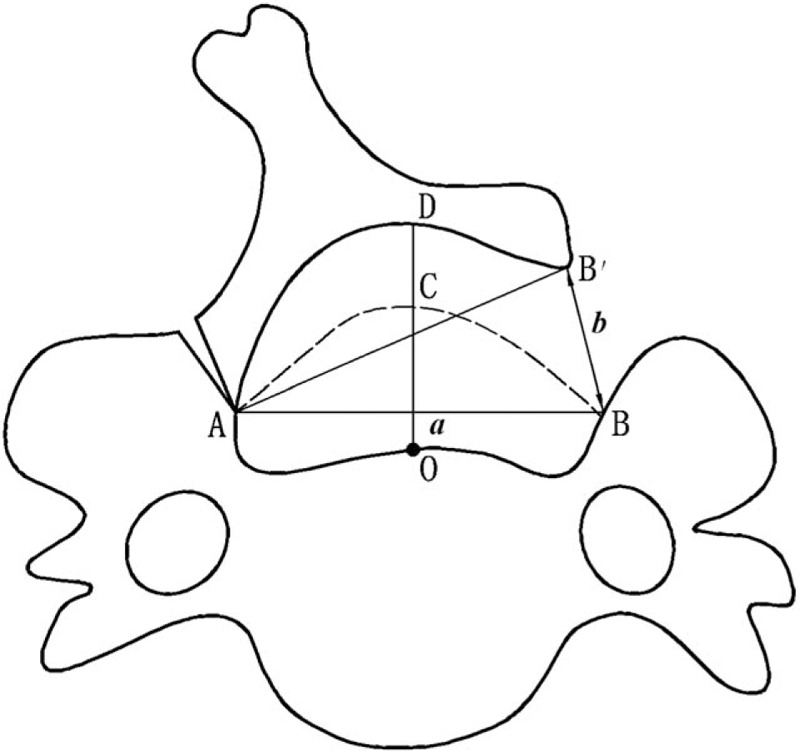
The geometric deformation of the cross-sectional area before and after the surgery operation. The dashed curve A-C-B represents the shape of the inner edge of the lamina before surgery, while the solid curve A-D-B′ is that after single-door cervical laminoplasty. Points A and B represent the most medial points of the bilateral laminar gutters. The distance of A and B is defined as the transverse canal diameter (TCD), represented by *a*. B and B′ represent the open sites of the lamina before and after surgery, respectively. The length of B-B′ is defined as the laminoplasty opening size (LOS), represented by *b*. These three key points form an isosceles triangle ΔBAB′. LOS = laminoplasty opening size, TCD = transverse canal diameter.

This study was mainly to analyze geometric deformation in the cervical canal measured before and after the surgery operation; a set of corresponding equation was derived to unveil the relationship between the actual LOS, the TCD, and the increment of the cross-sectional area. An example structure of SDCL is depicted in Figure 1. The dashed curve A-C-B represents the shape of the inner edge of the lamina before surgery, while the solid curve A-D-B’ is that after SDCL.

As shown in Figure 1A and B are the most medial points of the bilateral laminar gutters. In the present investigation, the distance between A and B was defined as TCD, represented by *a*. Besides, O in Figure 1 is the midpoint on the central symmetry line of the vertebral body. Straight line O-C stands for a vertical line intersecting the inner lamina edge at C before the surgery, whereas at D after the SDCL. Similarly, B and B′ represent the open sites of the lamina before and after surgery, respectively. Therefore, the distance between B and B′, represented by *b*, indicates the value of actual LOS.

**Figure 2:**
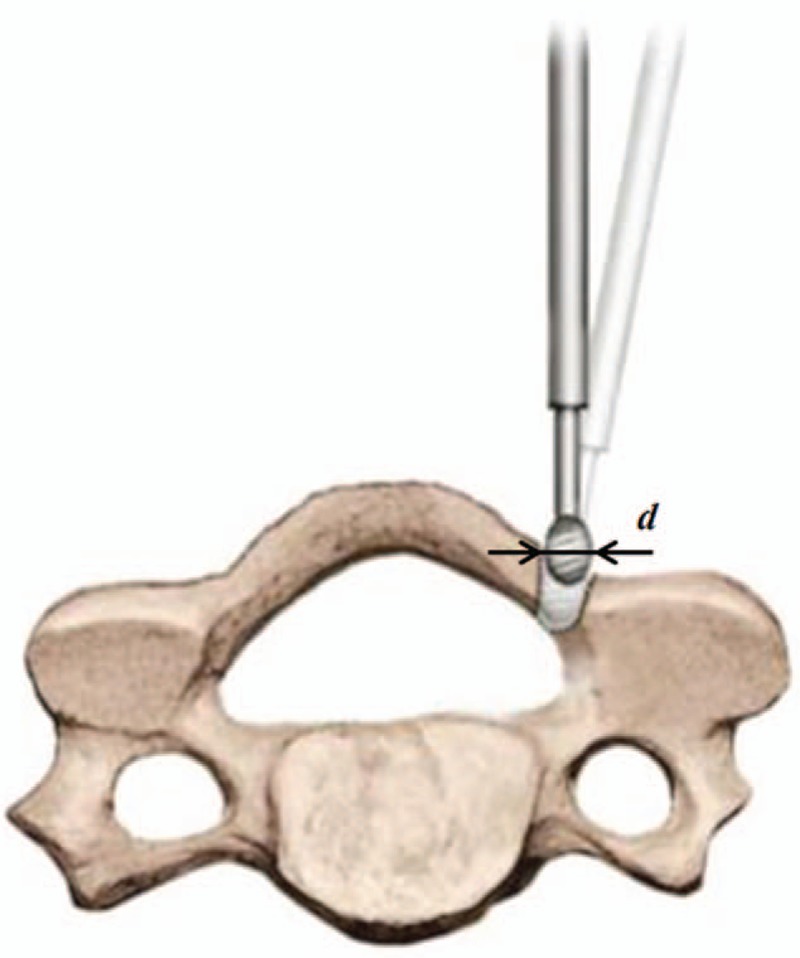
Preparing the lamina opening using a high-speed hand drill in single-door cervical laminoplasty. During the clinical operation, some lamina tissue would be removed by the drill bit. The size of “worn-off” lamina tissue depends on diameter of the drill bit, which was represented as *d* in the modeling.

It is worth noting that some lamina tissue would be removed when preparing the lamina opening at the practical SDCL operation (see Fig. 2). The size of this “worn-off” lamina depends on the surgery drill bit diameter, which was represented as *d* in the modeling. Also, the size of the mini-plate was represented as *c*. Therefore, the actual LOS would be the difference of the size of the mini-plate and the drill bit, namely *b* = *c*–*d*. Given the fact that the lamina was generally a rigid structure composed of bone and there was normally no significant flexible deformation during the procedure of operation, it was determined that AB=AB′= *a*. The manual comparison and analysis of the lamina's geometric shape before and after the surgery indicated that the size of the geometric area of Triangle ΔBAB′ was equal to the increment of cross-sectional area of the spinal canal. Additionally, it was geometrically derived in the isosceles triangle ΔBAB′ that 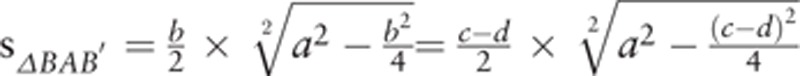 (*a* is the TCD, *b* the actual LOS, *c* the size of mini-plate, and *d* the diameter of the drill bit used during the surgery operation.)

### 2.3 Radiology

CT scans were conducted to visualize the samples with the 64 slice computed tomography scanner (GE Light Speed 64 slice VCT, GE Healthcare, Milwaukee, WI) for all the patients before and 1 week after their surgery. The scan started from C1 to C7. The scanning parameters includes: tube voltage of 120 kVp, tube current-time product of 220 mAs, section thickness of 0.625 mm, reconstruction interval of 0.625 mm, gantry rotation time of 0.5 seconds, pitch of 0.925, matrix of 512 × 512, and a FOV of 200 × 200 mm).

After scanning, the raw data were delivered to GE postprocessing workstation (ADW 4.4), where the scan data were reconstructed into axial, coronal and sagittal images. Axial CT cuts performed at each pedicle level from C3 to C7 were adopted for experimental measurements.

The picture archiving and communication system (PACS) was applied to measure the distance between Point A and B (refer to Fig. 1 for details), as well as the size of cross-sectional area before and after the surgery operation. Two of the authors performed the data measurements three times independently with a resolution of 0.01 mm or 0.01 mm^2^. The intraobserver and interobserver reliability with Cronbach alpha values are all higher than 0.85 in this project, which confirmed satisfactory consistence of all the manual measurement (see Table 1). The average values of their measurements on each variable were used for analysis variables in the project, in order to achieve a high standard of reliability.

**Table 1:**
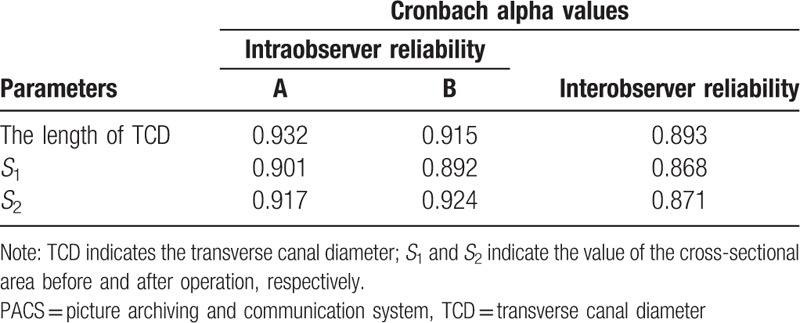
Intraobserver and interobserver reliability with Cronbach alpha values for 2 independent team members, A and B. The measurements were performed using PACS.

### 2.4 Statistical analysis

In this research, statistical analysis of the clinical data was performed with software SPSS verion24.0 (SPSS, Inc., Chicago, IL). The mean value and the standard deviation (SD) were derived for each measured variable at a significance level of *P* < .05. The differences between the results obtained by PACS and that estimated from the modeling equation were evaluated with the paired *t* test to verify the feasibility and reliability of the mathematical model.

## 3 Results

**Figure 3:**
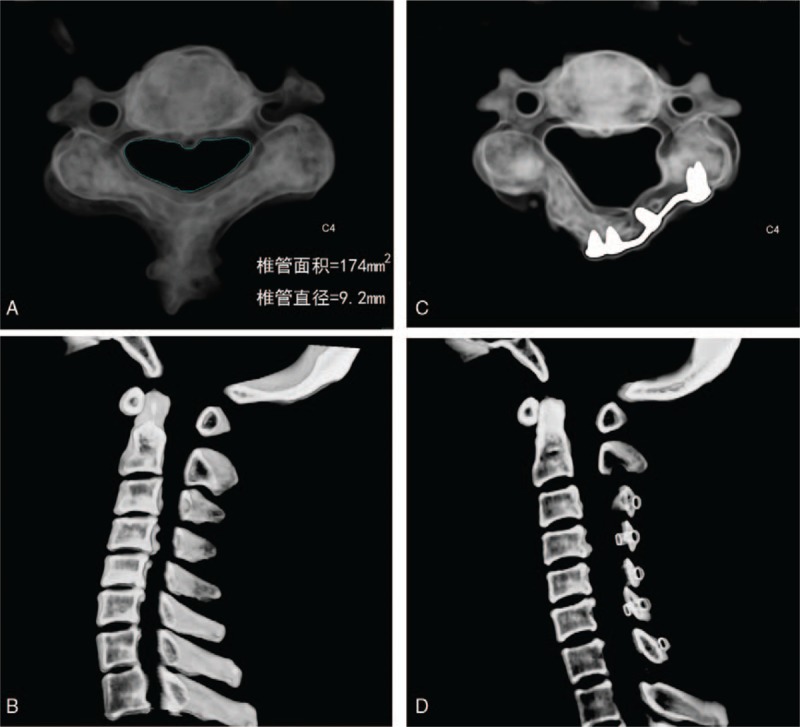
The CT scanning images before and after operation. (A) and (B) are the CT scanning images before the surgery operation; (C) and (D) are after the surgery.

In the present research, all the surgery operations were fully prepared and successfully conducted. The patients reported satisfactory recovery results after the operations (see Fig. 3). The experimental data were collected as above-mentioned before and after the surgery.

For each surgery operation, the research group measured the value of cross-sectional area before and after the surgery, represented by *S*_*1*_ and *S*_*2*_, respectively, and the TCD. The measurements were applied to calculate the increment of cross-sectional area Δ*S*_*2*_. Thus, the results were compared with the theoretical values estimated from the mathematical equation in the model.

After each surgery operation, the CT scans of cervical spine indicated that the cross-sectional area of spine canal was enlarged significantly. It was also discovered that there existed a significant variation of the cross-sectional area for each patient before and after his or her surgery in each specific segment (*P* < .01, see Table 2). However, there was no significant difference between the cross-sectional area value estimated from the modeling equation and that measured by the software for each individual segment (*P* > .05, see Table 3), which confirmed the high reliability of the present mathematical modeling.

**Table 2:**
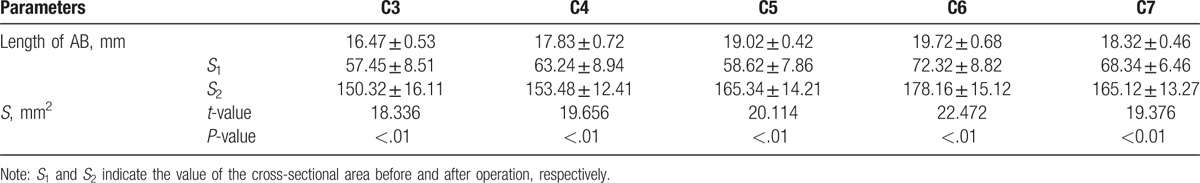
Pre- and postoperative parameters of C3–C7 of 25 patients for single open-door laminoplasty (n = 25).

**Table 3:**
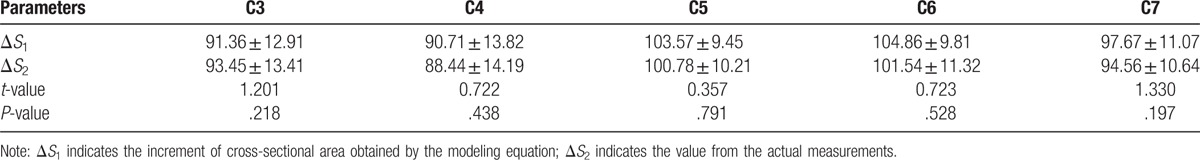
Comparison and statistic analysis of the increment of cross-sectional area obtained by the modeling equation and that from actual measurements using the paired *t* test (n = 25).

## 4 Discussion

For decades, the SDCL has been widely accepted as the optimum selection in clinical treatment of cervical spinal stenosis. The main reason is because of its reliable functionality and relatively straightforward operation procedure for operators.^[15,16]^ In the clinical operation, centerpiece mini-plate (Medtronic Inc., Minneapolis, Minnesota) was the primary choice for SDCL. The centerpiece mini-plate has been for years proved to be safe, reliable, and relatively simple in operation.^[17–19]^

Generally, the LOS plays a critical role in the SDCL for affecting the increment of cross-sectional area, further determines the effectiveness of the surgery. On the other hand, the LOS is mainly determined by the size of the mini-plate utilized in the operation. Therefore, the key variable for SDCL is the optimum selection of the size of the mini-plate for each individual surgery. Unfortunately, there has not been so far a widely accepted standard for the selection of mini-plate, which is essentially based on the experience of the surgery operator. It would potentially lead to undesired surgery treatments. The proposed mathematical modeling in this paper offered assistance for setting up such a standard that would enable the surgeon or operator to select the optimum mini-plate and drill bit for each individual surgery.

As mentioned in previous section and emphasized here, some lamina tissue would be removed when preparing the lamina opening using a hand drill during the clinical operation. For a practical instance, the size of lamina tissue removal would be in the range between 1.5 and 4 mm; the specific value for each operation depends on the drill bit diameter.^[20]^ In previous theoretical research, however, this removal of lamina tissue was normally neglected in the calculation.

Nevertheless, it was noticed in the present research that it would contribute to surgery errors when this “tissue loss” was dismissed. Herein, this small lamina tissue removal was taken into consideration to achieve higher accuracy and reliability of the surgery operation.

Given the fact that this lamina tissue loss was very critical for the preciseness of the surgery operation, a new variable was introduced in the present modeling. It was defined as the actual LOS, namely the difference of the mini-plate size and the drill bit diameter. Besides, note that the length of TCD was normally different from patient to patient. Therefore, it was not always feasible to estimate the increment of cross-sectional area based only on geometric derivation of the actual LOS. The influence of TCD on the cross-sectional area was also taken into account in the modeling. These detailed elements enabled the mathematical equations to be more accurate and clinically reliable.

Based on the geometric analysis of the deformation of spine canal before and after the surgery, a mathematical model was derived to reveal the correlation of actual LOS, the TCD, and the increment of cross-sectional area: 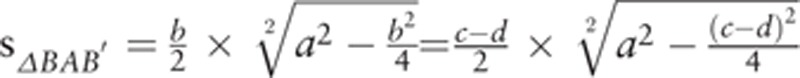. This equation was proved to be accurate with a high confident level for each segment of C3–C7.

It is worth mentioning that the most favorable cross-sectional area and the increment after the surgery were still under discussion in academic research. Whereas, the primary object of the current study is to propose a theoretical model based on the geometric analysis of the spine canal's structure before and after SDCL. According to this model, the target LOS would be estimated before the surgery depending on the expected increment of cross-sectional area of the spine canal. Note that the expected increment of cross-sectional area of the spine canal in the calculation would be any preferred value before the clinical surgery for each individual. Therefore, for each segment of the cervical spine, the convenient calculation would be performed for the surgery operator to select the optimum mini-plate and hand drill bit, with some simple measurements but much higher reliability. In a practical example, if the operator was seeking a specific increment value of cross-sectional area, the variables, such as the expected increment of cross-sectional area *S*_ΔBAB′_, the length of TCD *a*, and the diameter of the high-speed drill bit *d*, would be substituted into the equation to calculate the optimum size of mini-plate *c*; likewise, the variables including *c*, *a*, and *d* would be plugged into the equation to estimate the actual increment of S_ΔBAB’_, which turned out to be a reliable option to predict the effectiveness of the selected mini-plate before the surgery.

In this paper, a mathematical model was introduced to characterize the geometric relation of the clinical parameters of SDCL. With the assistance of this model, the measurements of CT scans would be undertaken to estimate a more accurate increment of cross-sectional area and an optimum mini-plate to select for the surgery operation. In another word, this model provided a more accurate quantitative reference to perform the clinical operation of SDCL, which would make the surgery more precise, effective, and reliable.

On the other hand, it is acknowledged that there are potential improvements to the present project. For instance, the lateral hinge is normally located at the medial border of the facet joints.^[11,12,21,22]^ Unfortunately, it is practically impossible to find the exact same position for all surgeries in the clinical operation, the experiment uncertainty would accumulate, even though all the procedures were performed by the same surgeon to reduce the experimental variance as much as possible in this research. Besides, the accuracy of the measurements would be influenced by other elements such as the deflection from the exact location of the CT scan and possible non-consistence of the cross-section at the measurements.

## 5 Author contributions

**Conceptualization:** W. Lei, Y. Zhang.

**Data curation:** R-J. Tian, S-B. Hu, X-J. Yang, X. Su, Y. Zhang.

**Funding acquisition:** Y. Zhang.

**Investigation:** X-J. Yang.

**Methodology:** X-J. Yang, X. Su.

**Project administration:** W. Lei.

**Resources:** R-J. Tian, S-B. Hu, X-J. Yang, X. Su.

**Software:** S-B. Hu.

**Supervision:** S-B. Hu, X-J. Yang, X. Su, Y. Zhang.

**Validation:** Y. Zhang.

**Visualization:** W. Lei.

**Writing – original draft:** R-J. Tian, S-B. Hu.

**Writing – original draft:** X-J. Yang.

## Acknowledgments

The authors are grateful for the permits to access the facilities of the Medical Imaging Research Center in the Department of Radiology of Xijing Hospital.
